# Environmental Action-Based Learning for Sustainability Competencies: EMKONTAN Model on Environmental Literacy, Creative Thinking, and Collaboration among Pre-Service Biology Teachers

**DOI:** 10.12688/f1000research.181913.2

**Published:** 2026-06-20

**Authors:** Nurwidodo Nurwidodo, Iin Hindun, Husamah Husamah

**Affiliations:** 1 Biology Education, Universitas Muhammadiyah Malang, Malang, East Java, 65144, Indonesia

**Keywords:** Action-based learning, collaboration skills, creative thinking, environmental literacy, pre-service biology teachers, sustainability education, teacher education

## Abstract

**Background:**

Sustainability transitions require educators who can translate environmental knowledge into creative, collaborative, and action-oriented learning. This study examined the effectiveness of the Environmental Mapping, Conservation, Action, and Evaluation learning model (EMKONTAN) in strengthening environmental literacy, creative thinking, and collaboration skills among pre-service biology teachers.

**Methods:**

A quasi-experimental pretest-posttest non-equivalent control group design was implemented in an environmental science course involving three learning conditions: EMKONTAN, problem-based learning, and regular instruction. Of 120 eligible students, 108 students with complete pretest and posttest data were included in the analysis. Environmental literacy was measured using an adapted environmental literacy instrument, creative thinking through essay-based tasks, and collaboration through an observation rubric. One-way analysis of covariance was used to compare adjusted posttest means after controlling for pretest scores, followed by least significant difference post hoc testing.

**Results:**

The EMKONTAN group achieved the highest adjusted means for environmental literacy (76.30), creative thinking (96.18), and collaboration (78.89). The learning model had statistically significant effects on environmental literacy, F(2,104) = 126.54, p < .001, partial eta squared = .709; creative thinking, F(2,104) = 274.51, p < .001, partial eta squared = .841; and collaboration, F(2,104) = 161.33, p < .001, partial eta squared = .756.

**Conclusions:**

These findings suggest that an environmental action-based learning cycle that integrates problem identification, field observation, action planning, implementation, monitoring, evaluation, and follow-up can support sustainability competencies in teacher education. The study contributes a contextual instructional model for preparing future biology teachers to design participatory environmental learning in developing-country higher education settings.

## Introduction

Environmental degradation, climate change, biodiversity decline, waste generation, and resource depletion increasingly demand educational responses that are not limited to knowledge transmission. ^1– 6^ Higher education institutions, especially teacher education programs, are expected to prepare graduates who can interpret environmental problems, design responsible actions, and guide learners toward sustainable practices. ^7– 9^ In biology teacher education, this expectation is especially important because future teachers will mediate scientific knowledge, environmental values, and pro-environmental behavior among school students. ^10– 14^

Sustainability education therefore requires the integration of environmental literacy, creative thinking, and collaboration. Environmental literacy enables learners to understand ecological systems, evaluate environmental issues, and make informed decisions. ^15, 16^Creative thinking supports the generation of alternative solutions when environmental problems are complex, contextual, and uncertain. ^17, 18^ Collaboration is equally essential because most sustainability challenges cannot be solved through isolated individual action. ^19, 20^ Together, these competencies align with the educational dimension of the Sustainable Development Goals and with the broader agenda of preparing citizens who can participate in sustainability transitions.

However, university environmental science courses are often still dominated by lectures, textbook-based tasks, and assessment formats that emphasize factual mastery. Such approaches may improve conceptual familiarity, but they are less likely to cultivate authentic inquiry, field-based sensitivity, collective responsibility, and action competence. ^21– 25^ Problem-based learning (PBL) has been widely used to situate learning around real problems, yet PBL is not always explicitly connected to environmental mapping, conservation-oriented action, monitoring, and follow-up programs. ^26, 27^This creates a pedagogical gap: sustainability-oriented teacher education needs a learning model that combines problem inquiry with concrete environmental action and reflective continuity.

The EMKONTAN model was developed to address this gap. In this study, EMKONTAN refers to an environmental action-based learning cycle consisting of socialization and problem identification, campus environmental observation and data collection, action planning with conservation integration, action implementation, monitoring and evaluation, and follow-up through student creativity programs. The model is designed to move students from awareness to evidence-based analysis, from analysis to collective action, and from action to reflection and continuation. ^28– 32^ This makes it suitable for sustainability education because it links scientific understanding with situated practice and social participation.

The present study aimed to test whether EMKONTAN is more effective than PBL and regular instruction in improving environmental literacy, creative thinking, and collaboration among pre-service biology teachers. The study tested three hypotheses: H1, students taught through EMKONTAN achieve higher environmental literacy than students taught through PBL and regular instruction after controlling for pretest scores; H2, students taught through EMKONTAN achieve higher creative thinking than students in the comparison groups; and H3, students taught through EMKONTAN achieve higher collaboration skills than students in the comparison groups. The study contributes empirical evidence on action-based sustainability learning in Indonesian higher education and provides an instructional model that can be adapted in biology teacher education programs.

## Methods

### Research design

This study used a quasi-experimental pretest-posttest non-equivalent control group design ( Table 1). Three intact classes were assigned to different learning conditions: an experimental class taught using EMKONTAN, a positive control class taught using PBL, and a negative control class taught using regular instruction. Pretests were administered before the intervention and posttests were administered after the learning sequence using comparable instruments for each outcome. The design was selected because the study was conducted in authentic university classes where random assignment of individual students was not feasible.

**Table 1.** Quasi-experimental research design.

Group	Pretest	Learning treatment	Posttest
Experimental group	O1	EMKONTAN environmental action-based learning	O2
Positive control group	O3	Problem-based learning	O4
Negative control group	O5	Regular instruction	O6

### Participants and setting

The study was conducted in an environmental science course for pre-service biology teachers in East Java, Indonesia. The eligible population consisted of 120 students enrolled in the relevant course. Before data collection, all participants were informed about the purpose of the study, the learning activities involved, the type of data to be collected, the voluntary nature of participation, and their right to withdraw from the study at any stage without academic penalty. Written informed consent for participation in the research was obtained from all participants before the pretest was administered. No minors were involved in this study. After data screening, 108 students with complete pretest and posttest records across the measured variables were included in the final analysis. Twelve cases were excluded because they did not have complete paired records for all measured variables, mainly due to absence during one of the testing sessions or incomplete observation records for the collaboration rubric. Complete-case analysis was applied consistently across the three outcome variables so that all ANCOVA models used the same analytic sample. Nevertheless, the loss of 12 eligible students may introduce attrition-related bias if the incomplete cases differed systematically from the retained cases; therefore, this issue is acknowledged in interpreting the findings and in the limitations.

### Learning treatments

The EMKONTAN class followed a six-stage environmental action cycle ( Table 2). Students first discussed the learning steps and identified environmental problems around the campus. They then conducted field observation and collected environmental data based on environmental maps. In the third stage, students prepared action plans and explored opportunities for conservation integration. The fourth stage required students to implement actions collaboratively, document the process, and produce project portfolios. The fifth stage involved monitoring, evaluation, presentation, and reflection. Finally, students designed follow-up activities that could be developed into student creativity programs related to environmental science topics.

**Table 2.** Operational syntax of the EMKONTAN learning model.

No.	EMKONTAN phase	Lecturer role	Student activity
1	Socialization and environmental problem identification	Introduces the learning steps, facilitates the selection of relevant environmental issues, and poses essential questions.	Express ideas, identify campus environmental problems, and map possible solutions.
2	Observation and environmental data collection	Guides collaborative field observation and data collection based on environmental maps.	Conduct observation, collect data, and document environmental conditions.
3	Action planning and conservation integration	Facilitates action planning and links solutions to conservation values.	Select solution strategies, prepare schedules, and formulate conservation-oriented action plans.
4	Action implementation	Monitors student action, provides guidance, and records important learning activities.	Implement environmental actions collaboratively and prepare project portfolios.
5	Monitoring and evaluation	Assesses process and product achievement and evaluates sustainability potential.	Present group outputs, written reports, and project products.
6	Follow-up program	Guides students in planning continuity through student creativity programs.	Prepare follow-up programs based on environmental science topics.

The PBL class was implemented as a positive comparison condition using a general problem-oriented learning sequence. Students were introduced to contextual environmental problem scenarios prepared by the lecturer, worked in small groups to clarify the problem, identified what they needed to learn, searched for relevant information from textbooks and other learning resources, discussed alternative solutions, and presented their proposed answers to the class. The lecturer acted mainly as a facilitator by asking guiding questions, monitoring group discussion, and providing feedback on the feasibility of proposed solutions. Unlike EMKONTAN, the PBL condition did not require students to conduct systematic environmental mapping, implement a conservation-oriented action project, monitor action outcomes, or prepare follow-up student creativity programs. The regular instruction class was used as a negative comparison condition and consisted of lecturer explanation, textbook-based discussion, question-and-answer sessions, and individual assignments related to environmental science concepts. Students in this group were not required to complete structured field observation, collaborative action implementation, project portfolios, or monitoring and follow-up activities. Thus, the comparison was intended to distinguish the added value of the EMKONTAN cycle from both a general problem-oriented approach and conventional instruction.

### Instruments

The research instruments and measured indicators are summarized in Table 3. Environmental literacy was measured using an adapted environmental literacy instrument based on dimensions of ecological knowledge, issue investigation and analysis, environmental sensitivity, and pro-environmental behavior. Creative thinking was measured through essay-based tasks assessed using indicators of curiosity, fluency, originality, flexibility, elaboration, and divergent thinking. Collaboration skills were evaluated using an observation rubric covering productive work, respect, compromise, and shared responsibility. The creative thinking and collaboration rubrics used a four-level scoring framework adapted from 21st-century skill assessment practices.

**Table 3.** Research instruments and measured indicators.

Outcome	Instrument	Main indicators	Score interpretation
Environmental literacy	Adapted environmental literacy instrument	Ecological knowledge, issue investigation, analysis, sensitivity, pro-environmental behavior	Higher score indicates stronger environmental literacy.
Creative thinking	Essay-based test with rubric	Curiosity, fluency, originality, flexibility, elaboration, divergent thinking	1 = beginner, 2 = basic, 3 = proficient, 4 = advanced.
Collaboration	Observation rubric	Productive work, respect, compromise, shared responsibility	1 = poor, 2 = fair, 3 = good, 4 = very good.

### Data analysis

Data were analyzed using descriptive statistics and inferential analysis. Normality was examined using the Kolmogorov-Smirnov test, and homogeneity of variance was examined using Levene’s test. One-way analysis of covariance (ANCOVA) was used to compare posttest outcomes among the three learning models while controlling for pretest scores. Least significant difference post hoc testing was used to identify pairwise differences in adjusted means. Statistical testing used a significance level of 0.05. ANCOVA was retained because it directly corresponded to the pretest-posttest non-equivalent control group design and allowed baseline differences to be statistically controlled. In addition, each group contained more than 30 students, and ANCOVA is generally considered relatively robust to moderate departures from normality when sample sizes are reasonably balanced. However, because the normality test indicated non-normal distributions and the collaboration variable showed unequal variance, the findings should be interpreted as strong but not assumption-free evidence. The interpretation therefore emphasizes the consistency of the adjusted mean pattern and the very large effect sizes, while recommending that future studies complement ANCOVA with robust, rank-based, or non-parametric sensitivity analyses.

## Results

### Assumption checks

The Kolmogorov-Smirnov test indicated that the pretest and posttest distributions for the measured variables were not normally distributed (p < .001) ( Table 4). Levene’s test showed that environmental literacy and creative thinking met the homogeneity assumption, whereas collaboration did not. These results suggest that the ANCOVA findings are informative but should be interpreted cautiously, particularly for collaboration. However, the three outcomes showed the same directional pattern in the adjusted means, and the partial eta squared values were very large. Therefore, the results are presented as evidence of strong practical differences among learning conditions, while the assumption violations are explicitly considered in the Discussion and limitations. The complete-case sample used in the inferential analysis was N = 108.

**Table 4.** Summary of homogeneity testing.

Variable	Levene F	df1	df2	p-value	Interpretation
Environmental literacy	2.521	2	105	.085	Homogeneous variance
Creative thinking	0.667	2	105	.516	Homogeneous variance
Collaboration	19.280	2	105	< .001	Variance heterogeneity

### Environmental literacy

The learning model significantly affected environmental literacy after controlling for pretest scores, F(2,104) = 126.539, p < .001, partial eta squared = .709. The EMKONTAN group obtained the highest adjusted mean (76.301), followed by PBL (45.513) and regular instruction (42.852). The EMKONTAN group improved by 77.31%, whereas PBL showed a smaller increase of 9.54% and regular instruction showed a slight decline of 2.54%.

### Creative thinking

The learning model also had a significant effect on creative thinking, F(2,104) = 274.512, p < .001, partial eta squared = .841. The EMKONTAN group achieved the highest adjusted mean (96.176), followed by PBL (81.939) and regular instruction (42.247). This pattern indicates that both problem-oriented learning and environmental action-based learning supported creative thinking, but the explicit action cycle in EMKONTAN produced the strongest result.

### Collaboration skills

For collaboration skills, the learning model had a significant effect, F(2,104) = 161.325, p < .001, partial eta squared = .756. The adjusted mean for EMKONTAN was 78.89, compared with 46.19 for PBL and 16.12 for regular instruction ( Table 5). Although the homogeneity assumption was not met for this variable, the magnitude and consistency of the descriptive pattern suggest that EMKONTAN provided richer opportunities for shared responsibility, compromise, respectful interaction, and productive group work.

**Table 5.** Descriptive and adjusted outcome scores by learning model.

Outcome	Group	Pretest mean	Posttest mean	Adjusted mean	Post hoc notation	Gain
Environmental literacy	EMKONTAN	43.08	76.39	76.30	a	77.31%
	PBL	40.75	44.64	45.51	b	9.54%
	Regular	44.78	43.64	42.85	b	−2.54%
Creative thinking	EMKONTAN	35.22	96.36	96.18	a	173.58%
	PBL	45.50	81.75	81.94	b	79.67%
	Regular	40.22	42.25	42.25	c	5.04%
Collaboration	EMKONTAN	11.31	78.94	78.89	a	598.28%
	PBL	13.08	46.19	46.19	b	253.08%
	Regular	15.53	16.06	16.12	c	3.40%

**Figure 1.**
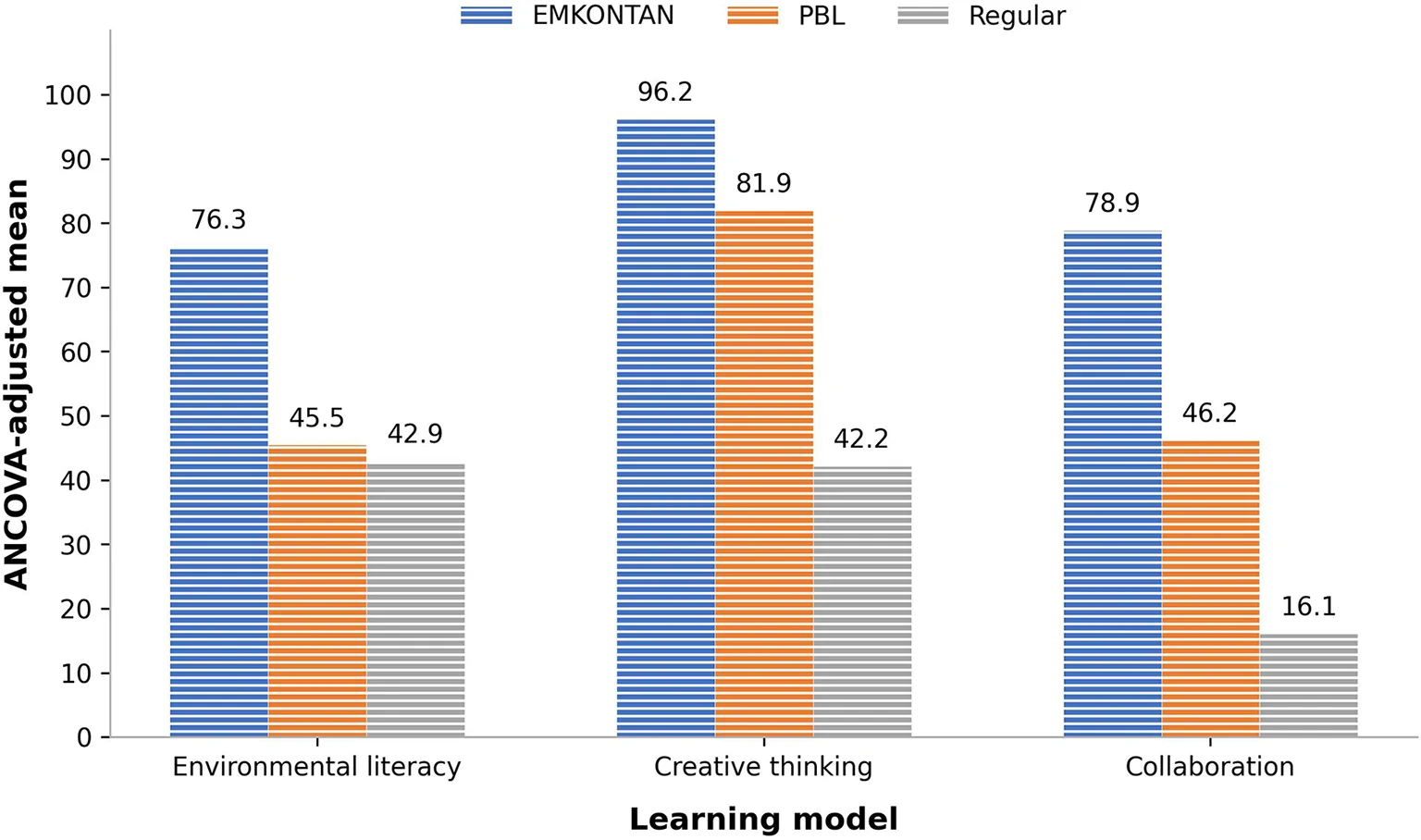
ANCOVA-adjusted posttest means across the three measured sustainability competencies.

**Figure 2.**
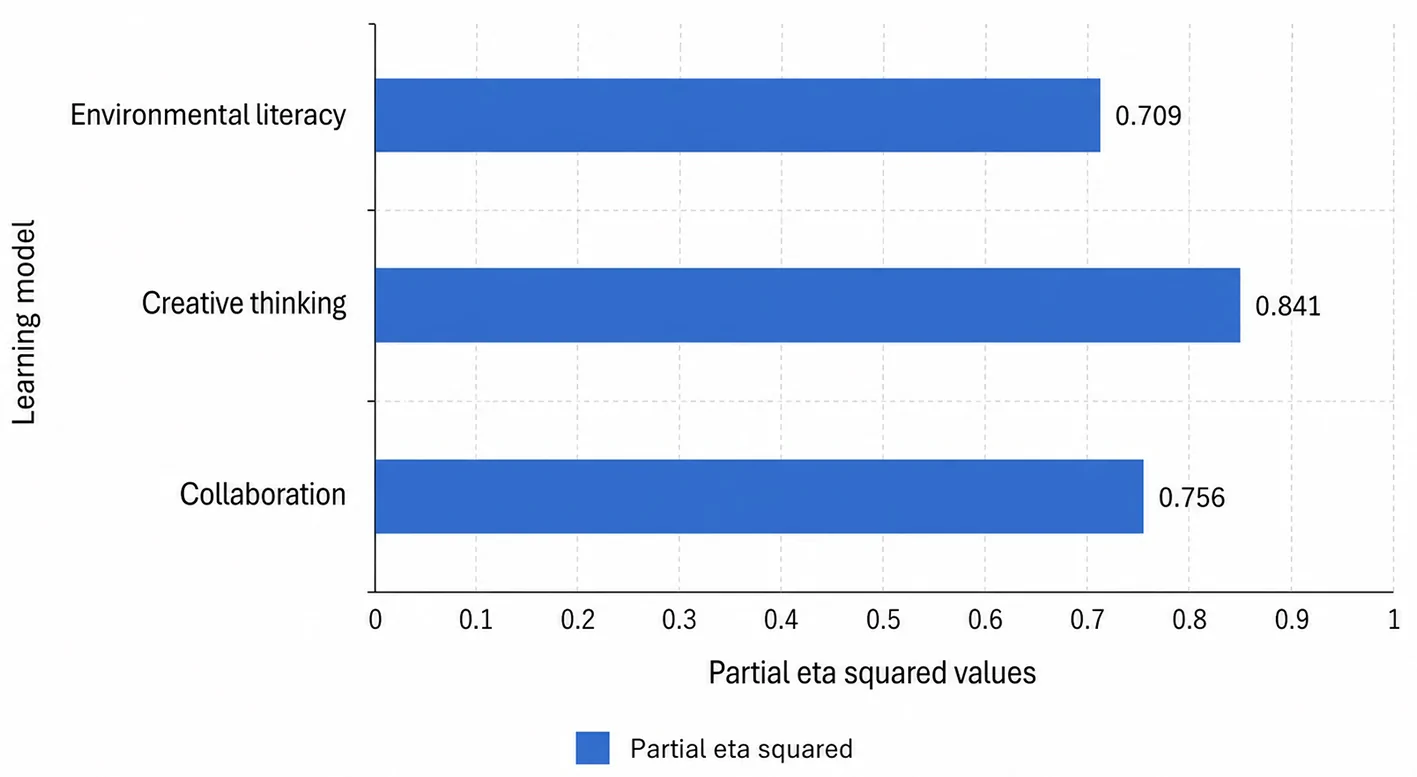
Partial eta squared values for the effect of learning model on each outcome.

Table 6 summarizes the ANCOVA results, while Figure 1 displays the adjusted posttest means and Figure 2 shows the magnitude of the learning model effects. Across all three outcomes, the learning model effect was statistically significant and practically large. EMKONTAN consistently produced the highest adjusted means, followed by PBL and regular instruction. The largest effect was found for creative thinking, followed by collaboration and environmental literacy. Because the same comparative pattern appeared across descriptive scores, adjusted means, and effect sizes, the results support the conclusion that EMKONTAN was associated with stronger sustainability-related outcomes than the two comparison conditions. At the same time, the collaboration result should be read cautiously because the homogeneity assumption was not met.

**Table 6.** Summary of ANCOVA results.

Outcome	Corrected model F	Learning model F	p-value	Partial eta squared	R2	Adjusted R2
Environmental literacy	96.458	126.539	< .001	.709	.736	.728
Creative thinking	186.169	274.512	< .001	.841	.843	.838
Collaboration	127.249	161.325	< .001	.756	.786	.780

The ANCOVA results indicate that the learning model had a statistically significant effect on all three outcome variables: environmental literacy, creative thinking, and collaboration. As shown in Table 6, the corrected model was significant for environmental literacy, F = 96.458, p < .001; creative thinking, F = 186.169, p < .001; and collaboration, F = 127.249, p < .001. These results confirm that, after controlling for pretest scores, the type of learning model contributed significantly to differences in students’ posttest performance across the three measured competencies.

More specifically, the learning model effect was significant for environmental literacy, F = 126.539, p < .001, with a partial eta squared value of .709. This indicates a very large effect, suggesting that 70.9% of the explainable variance in adjusted environmental literacy scores was associated with differences in the learning model. The model also explained a substantial proportion of variance, with R ^2^ = .736 and adjusted R ^2^ = .728. As illustrated in Figure 1, the EMKONTAN group obtained the highest ANCOVA-adjusted mean score for environmental literacy (76.3), followed by the PBL group (45.5) and the regular learning group (42.9). This pattern indicates that EMKONTAN was more effective than both comparison models in strengthening students’ environmental literacy.

A similar pattern was found for creative thinking. The effect of the learning model was statistically significant, F = 274.512, p < .001, with the largest effect size among the three outcomes, partial eta squared = .841. This means that 84.1% of the explainable variance in adjusted creative thinking scores was related to the learning model used. The overall model also showed strong explanatory power, with R ^2^ = .843 and adjusted R ^2^ = .838. The adjusted mean scores show that EMKONTAN produced the highest creative thinking score (96.2), followed by PBL (81.9), while regular learning resulted in a much lower score (42.2). These findings suggest that EMKONTAN was particularly powerful in developing students’ creative thinking, especially when compared with regular instruction.

For collaboration skills, the ANCOVA also revealed a significant learning model effect, F = 161.325, p < .001, with a large effect size, partial eta squared = .756. The model explained 78.6% of the variance in collaboration outcomes, with an adjusted R ^2^ of.780. The adjusted mean scores further demonstrate that EMKONTAN led to the highest collaboration performance (78.9), followed by PBL (46.2), while regular learning showed the lowest adjusted mean score (16.1). This result indicates that the structured stages of EMKONTAN, especially observation, environmental problem analysis, action planning, project implementation, monitoring, and follow-up activities, provided stronger opportunities for students to practice productive teamwork, shared responsibility, and collaborative problem solving.

The findings consistently show that EMKONTAN outperformed both PBL and regular learning across all measured outcomes. The largest effect was observed in creative thinking, followed by collaboration and environmental literacy. The high partial eta squared values and adjusted R ^2^ values indicate that the learning model was not only statistically significant but also practically meaningful. These results support the conclusion that EMKONTAN is an effective sustainability-oriented learning model for improving environmental literacy, creative thinking, and collaboration among prospective biology teachers.

## Discussion

The findings support all three hypotheses. EMKONTAN produced the strongest gains in environmental literacy, creative thinking, and collaboration compared with PBL and regular instruction. The pattern suggests that sustainability competencies are more effectively developed when students engage in a complete learning cycle that begins with real environmental problems and proceeds toward action, monitoring, reflection, and follow-up. This is consistent with the premise that sustainability education must connect knowledge with situated problem solving and collective participation. ^33– 35^

The strong effect on environmental literacy can be explained by the combination of observation, data collection, and action planning. Students did not only receive environmental concepts; they had to identify environmental problems in their surroundings, collect evidence, analyze possible causes, and formulate feasible solutions. Such activities likely strengthened the link between ecological knowledge, environmental sensitivity, and pro-environmental behavior. The EMKONTAN cycle therefore supported environmental literacy as an integrated competence rather than as isolated factual knowledge.

The effect on creative thinking was also substantial. Creativity in sustainability education is not merely the production of unusual ideas; it involves generating workable alternatives for complex and context-dependent problems. ^17, 36, 37^ EMKONTAN required students to develop action plans, adapt strategies to field conditions, explore conservation integration, and revise ideas during monitoring and evaluation. These activities are aligned with fluency, flexibility, originality, elaboration, and divergent thinking. The PBL group also improved, which confirms the value of problem-oriented learning, but EMKONTAN produced higher scores because students moved beyond discussion into concrete action and reflective continuation.

Collaboration showed the largest relative gain in the EMKONTAN class. This result is pedagogically meaningful because the model required students to divide roles, negotiate decisions, implement actions, document progress, present outputs, and respond to peer feedback. Collaboration was therefore embedded in the core task structure, not treated as an optional group arrangement. The contrast with regular instruction indicates that collaboration skills are difficult to develop when learning relies mainly on individual assignments and teacher-centered explanation.

The study also highlights the importance of aligning sustainability education with teacher preparation. Pre-service biology teachers need to experience environmental learning that they can later adapt for school contexts. By participating in environmental mapping, conservation-oriented action, and follow-up planning, students gained not only content knowledge but also an instructional repertoire for designing participatory environmental learning. ^38– 40^ In this sense, EMKONTAN contributes to sustainability transitions through a teacher education pathway: strengthening the competencies of future teachers who may later influence students, schools, and communities.

The large effects should also be interpreted with a balanced view of possible alternative explanations. Because intact classes were used, differences in prior motivation, lecturer-student interaction, peer dynamics, group leadership, or familiarity with project-based tasks may have contributed to the observed outcomes. The novelty of the EMKONTAN activities may also have increased student engagement during the intervention. In addition, collaboration was assessed through observation, so evaluator expectations and the visibility of group performance could have influenced scoring despite the use of a rubric. These possibilities do not negate the positive findings, but they indicate that the effect sizes should be understood as evidence from an authentic classroom implementation rather than as definitive causal estimates from a fully randomized trial.

Despite these strengths, several methodological limitations should be linked directly to the interpretation of the findings. First, the quasi-experimental design used intact classes; therefore, the results support a strong association between EMKONTAN and improved outcomes but should not be interpreted as conclusive evidence of causality at the individual-student level. Second, the analysis included 108 complete cases from 120 eligible students. Although the complete-case criterion was applied consistently, incomplete data may have introduced bias if excluded students had different attendance, motivation, or performance profiles. Third, the non-normal distributions and the heterogeneous variance for collaboration mean that the ANCOVA results, especially for collaboration, should be interpreted with caution. The very large effect sizes strengthen the practical relevance of the findings, but they do not eliminate the need for sensitivity analyses using robust or non-parametric approaches. Fourth, the PBL and regular instruction conditions were designed as meaningful comparison groups, but differences in task authenticity, field engagement, and product requirements may have increased student motivation in the EMKONTAN group. Fifth, the study measured immediate post-intervention outcomes in one institutional context; therefore, generalizability to other universities, courses, or long-term teaching practice remains limited. Future studies should document attrition more fully, use randomized or matched designs where feasible, report robust sensitivity tests and confidence intervals, and examine retention and transfer into school-based teaching practice.

## Conclusion

This study demonstrates that EMKONTAN, an environmental action-based learning model, significantly improved environmental literacy, creative thinking, and collaboration skills among pre-service biology teachers than PBL and regular instruction. Compared with the two comparison conditions, EMKONTAN produced the highest adjusted posttest means and large effect sizes across all measured outcomes. The results indicate that sustainability competencies are strengthened when students are guided through a complete cycle of problem identification, environmental observation, action planning, action implementation, monitoring and evaluation, and follow-up program design. Because the study used intact classes, complete-case analysis, and ANCOVA under some assumption violations, the findings should be interpreted as strong classroom-based evidence that requires confirmation through replication and robust sensitivity analyses. The model is therefore promising for biology teacher education and for higher education courses that aim to connect environmental science learning with sustainability-oriented practice.

Future research should replicate the study in different institutions, use randomized or matched designs where possible, apply robust statistical sensitivity tests, and examine whether EMKONTAN-trained pre-service teachers can transfer these competencies into teaching practice during field experience or school internships. Longitudinal studies are also needed to determine whether environmental literacy, creativity, and collaboration are sustained after the course ends.

## Ethical considerations

The study involved human participants in an educational setting. Ethical approval was approved by Research Ethics Commission, Bureau of Research, Community Service, and Cooperation, University of Muhammadiyah Malang (approval number: E.5.b/119-RPK-UMM/IX/2025; date: 2 September 2025). Before data collection, all participants received information about the purpose of the study, the learning procedures, the data to be collected, confidentiality, voluntary participation, and the right to withdraw from the study at any time without academic consequences. Written informed consent was obtained from all participants prior to their involvement in the study. No minors were involved in this research.

## Data Availability

The underlying data supporting the findings of this study are openly available in Zenodo at https://zenodo.org/records/20055341, with the DOI: https://doi.org/10.5281/zenodo.20055341. ^41^ This dataset includes the anonymized pretest and posttest scores for environmental literacy, creative thinking, and collaboration skills; group allocation data for the EMKONTAN, problem-based learning, and regular instruction groups; the values used to calculate means, standard deviations, adjusted means, gain scores, ANCOVA results, and post hoc comparisons; and the data used to generate the figures and tables reported in this article. The extended data are openly available in Zenodo at https://zenodo.org/records/20055341 with the DOI: https://doi.org/10.5281/zenodo.20055341. ^41^ The extended data include the research instruments, scoring rubrics, learning treatment descriptions, and supporting materials required to understand and replicate the study procedures. Data are available under Creative Commons Attribution 4.0 International (CC BY 4.0). Not applicable.
