# Supplementary material for: Environmental Action-Based Learning for Sustainability Competencies: EMKONTAN Model on Environmental Literacy, Creative Thinking, and Collaboration among Pre-Service Biology Teachers
Source: F1000Res. 2026 Oct 13;15:777. Originally published 2026 May 22. [Version 3] doi: 10.12688/f1000research.181913.3 (PMC13234552; doi:10.12688/f1000research.181913.3)
Supplement: Peer review history

# Reviewer report: Reviewer response for version 3

**DOI:** 10.5256/f1000research.211788.r529696

**Published:** 2026-11-19

**Authors:** Ahmad Adnan Mohd Shukri

**Affiliations:** 1 Universiti Sains Malaysia, Penang, Malaysia

I have reviewed Version 3 and the authors’ responses. The revisions satisfactorily address the comments from both reviewers. The manuscript now explains more clearly how EMKONTAN differs from related teaching approaches and why environmental literacy, creative thinking, and collaboration were examined together. The practical examples also make the model easier to apply in biology teacher education.

 I appreciate the authors’ care in explaining the incomplete assessment records and interpreting the large effect sizes and ANCOVA findings with appropriate caution. The revised manuscript makes a valuable contribution to sustainability-oriented biology teacher education. I approve Version 3. No further revisions are required.

Is the work clearly and accurately presented and does it cite the current literature?

Yes

If applicable, is the statistical analysis and its interpretation appropriate?

Yes

Are all the source data underlying the results available to ensure full reproducibility?

Yes

Is the study design appropriate and is the work technically sound?

Yes

Are the conclusions drawn adequately supported by the results?

Yes

Are sufficient details of methods and analysis provided to allow replication by others?

Yes

Reviewer Expertise:

STEM Education; Science Education; Biology Education; Creative Thinking Skills and Styles; Critical Thinking Skills and Dispositions.

I confirm that I have read this submission and believe that I have an appropriate level of expertise to confirm that it is of an acceptable scientific standard.

# Reviewer report: Reviewer response for version 3

**DOI:** 10.5256/f1000research.211788.r529695

**Published:** 2026-11-19

**Authors:** Nasrul Hakim

**Affiliations:** 1 State Islamic Institute Metro, Metro, Indonesia

I have reviewed Version 3 and the authors’ point-by-point responses. The authors have addressed my ten comments and the other reviewer’s concerns. I am pleased to approve this version. No further revisions are required.

 The revised Introduction states the research gap more precisely, explains why environmental literacy, creative thinking, and collaboration were examined together, and connects the stages of EMKONTAN to the study hypotheses. It also clarifies what distinguishes EMKONTAN from related active-learning approaches and what the comparison with problem-based learning and regular instruction adds to earlier EMKONTAN research.

 I particularly appreciate the more nuanced interpretation of the large effect sizes. The Discussion explains how the alignment between learning activities and assessment tasks may have contributed to the observed differences, especially where students had more opportunities to practice and demonstrate collaboration. The manuscript also explains why 108 of the 120 eligible students were included in the analysis and acknowledges the potential for attrition bias and the need for caution when interpreting the ANCOVA results, particularly for collaboration.

 The concrete examples for environmental science, ecology, conservation biology, and biology teaching methods make the manuscript more applicable to teacher education. The closing synthesis brings the pedagogical contribution into focus by showing how environmental inquiry, authentic action, collaboration, monitoring, and follow-up fit within a continuous learning cycle. I appreciate the authors’ careful engagement with both reviews and consider the revised manuscript a valuable contribution to sustainability-oriented biology teacher education.

Is the work clearly and accurately presented and does it cite the current literature?

Yes

If applicable, is the statistical analysis and its interpretation appropriate?

Yes

Are all the source data underlying the results available to ensure full reproducibility?

Partly

Is the study design appropriate and is the work technically sound?

Yes

Are the conclusions drawn adequately supported by the results?

Yes

Are sufficient details of methods and analysis provided to allow replication by others?

Partly

Reviewer Expertise:

biology, sustainability

I confirm that I have read this submission and believe that I have an appropriate level of expertise to confirm that it is of an acceptable scientific standard.

# Reviewer report: Reviewer response for version 2

**DOI:** 10.5256/f1000research.203298.r495346

**Published:** 2026-09-30

**Authors:** Nasrul Hakim

**Affiliations:** 1 State Islamic Institute Metro, Metro, Indonesia

1. **The research gap in the Introduction could be stated more explicitly.** The authors are encouraged to clarify what previous studies on problem-based learning, environmental education, or action-based learning have not yet addressed, particularly regarding the simultaneous development of environmental literacy, creative thinking, and collaboration.
2. **The theoretical relationship among environmental literacy, creative thinking, and collaboration could be explained more clearly.** A brief explanation of why these three competencies were selected together as sustainability-related competencies would strengthen the conceptual foundation of the study.
3. **The novelty of the EMKONTAN model should be differentiated more clearly from other active-learning approaches.** The authors may briefly explain how EMKONTAN differs from problem-based learning, project-based learning, or experiential learning, especially in terms of environmental mapping, direct action, monitoring, evaluation, and follow-up activities.
4. **The transition from the research gap to the hypotheses could be strengthened.** Before presenting H1–H3, the authors may briefly explain why the specific characteristics of EMKONTAN are theoretically expected to influence environmental literacy, creative thinking, and collaboration.
5. **The Discussion would benefit from stronger engagement with previous empirical studies.** For each major finding, the authors are encouraged to indicate whether the results support, extend, or differ from earlier studies related to environmental education, sustainability education, PBL, or action-based learning.
6. **Some interpretations in the Discussion could be phrased more cautiously.** Given the quasi-experimental design, expressions such as “may have contributed to,” “was associated with,” or “may help explain” would be preferable to strongly causal statements.
7. **The very large effect sizes deserve slightly deeper interpretation.** The authors may briefly consider whether the magnitude of the effects could partly reflect the close alignment between EMKONTAN learning activities and the competencies being assessed, especially for creative thinking and collaboration.
8. **The interpretation of the collaboration findings could be refined.** Since the EMKONTAN group had substantially more opportunities to demonstrate collaborative behavior, the authors may acknowledge that the higher scores could reflect both competency development and greater opportunities to display the assessed behaviors.
9. **The practical implications for pre-service biology teacher education could be made more concrete.** The authors may briefly explain how EMKONTAN could be implemented in environmental science, ecology, conservation biology, or teaching-method courses.
10. **A short synthesis paragraph at the end of the Discussion would improve the manuscript.** Rather than repeating the statistical results, the authors could summarize the main pedagogical contribution of the study, particularly how the integration of environmental inquiry, authentic action, collaboration, monitoring, and follow-up may support sustainability-oriented teacher education.

Is the work clearly and accurately presented and does it cite the current literature?

Yes

If applicable, is the statistical analysis and its interpretation appropriate?

Yes

Are all the source data underlying the results available to ensure full reproducibility?

Partly

Is the study design appropriate and is the work technically sound?

Yes

Are the conclusions drawn adequately supported by the results?

Yes

Are sufficient details of methods and analysis provided to allow replication by others?

Partly

Reviewer Expertise:

biology, sustainability

I confirm that I have read this submission and believe that I have an appropriate level of expertise to confirm that it is of an acceptable scientific standard.

# Response

**Published:** 2026-09-30

**Authors:** Nurwidodo Nurwidodo

**Affiliations:** Biology Education, Universitas Muhammadiyah Malang, Malang, East Java, Indonesia

We sincerely thank the reviewer for these constructive and insightful comments. We have carefully revised the Introduction and Discussion to clarify the conceptual foundation, research gap, novelty of the EMKONTAN model, interpretation of the findings, and practical implications. Our point-by-point responses are provided below.

**Comment 1. The research gap in the Introduction could be stated more explicitly. The authors are encouraged to clarify what previous studies on problem-based learning, environmental education, or action-based learning have not yet addressed, particularly regarding the simultaneous development of environmental literacy, creative thinking, and collaboration.**

**Response:** Thank you for this important suggestion. We have revised the Introduction to state the research gap more explicitly. The revised text now explains that previous studies on problem-based learning, environmental education, and active-learning approaches have generally addressed selected cognitive, environmental, or interpersonal outcomes, whereas comparatively less attention has been given to learning designs that deliberately integrate environmental inquiry, authentic conservation action, monitoring, evaluation, and follow-up while simultaneously examining environmental literacy, creative thinking, and collaboration. We also acknowledge previous EMKONTAN studies and clarify that the present study extends this work by providing comparative evidence against both PBL and regular instruction across the three competencies within the same learning context.

**Comment 2. The theoretical relationship among environmental literacy, creative thinking, and collaboration could be explained more clearly. A brief explanation of why these three competencies were selected together as sustainability-related competencies would strengthen the conceptual foundation of the study.**

**Response:** We agree. A new paragraph has been added to the Introduction explaining the complementary relationship among the three competencies. Environmental literacy provides the knowledge, sensitivity, and evaluative capacity needed to understand environmental problems; creative thinking supports the generation and refinement of alternative solutions; and collaboration enables ideas to be negotiated, implemented, and evaluated through collective action. We further clarify why their simultaneous development is particularly relevant to sustainability-oriented pre-service biology teacher education.

**Comment 3. The novelty of the EMKONTAN model should be differentiated more clearly from other active-learning approaches. The authors may briefly explain how EMKONTAN differs from problem-based learning, project-based learning, or experiential learning, especially in terms of environmental mapping, direct action, monitoring, evaluation, and follow-up activities.**

**Response:** Thank you. We have added a paragraph that explicitly differentiates EMKONTAN from related active-learning approaches. The revised text acknowledges its conceptual overlap with PBL, project-based learning, and experiential learning while clarifying that the distinctive feature of EMKONTAN is the organization of environmental mapping, field evidence collection, conservation-oriented action, monitoring, evaluation, and follow-up as integral stages of a continuous environmental action cycle. We have intentionally framed the novelty as an integration and structuring of established active-learning principles rather than claiming that these principles are entirely unique to EMKONTAN.

**Comment 4. The transition from the research gap to the hypotheses could be strengthened. Before presenting H1–H3, the authors may briefly explain why the specific characteristics of EMKONTAN are theoretically expected to influence environmental literacy, creative thinking, and collaboration.**

**Response:** We agree and have added a bridging paragraph immediately before the study aim and hypotheses. The revision explains how environmental mapping, field observation, and evidence-based problem analysis may support environmental literacy; how action planning, generation of alternative solutions, implementation, and reflection may support creative thinking; and how shared data collection, division of responsibilities, implementation, monitoring, and follow-up provide repeated opportunities for collaboration. This provides a clearer theoretical rationale for H1–H3.

**Comment 5. The Discussion would benefit from stronger engagement with previous empirical studies. For each major finding, the authors are encouraged to indicate whether the results support, extend, or differ from earlier studies related to environmental education, sustainability education, PBL, or action-based learning.**

**Response:** Thank you for this recommendation. We have strengthened the Discussion for each outcome. The environmental literacy finding is now explicitly connected with previous research emphasizing issue investigation, situated environmental learning, and action-oriented environmental education. The creative-thinking finding is discussed in relation to previous problem-based, project-oriented, and creative problem-solving research, and we clarify how the present findings may extend this literature through the addition of field action, monitoring, and reflective continuation. The collaboration finding is now linked more clearly to literature emphasizing interdependent tasks, collective responsibility, and collaborative sustainability action.

**Comment 6. Some interpretations in the Discussion could be phrased more cautiously. Given the quasi-experimental design, expressions such as “may have contributed to,” “was associated with,” or “may help explain” would be preferable to strongly causal statements.**

**Response:** We agree. We reviewed the Results, Discussion, and Conclusion and revised several causal expressions. Statements such as “EMKONTAN improved,” “produced,” or “was more effective in developing” have been replaced, where appropriate, with more cautious formulations such as “was associated with,” “may have supported,” “may help explain,” or “the EMKONTAN condition showed higher adjusted scores.” These changes are consistent with the quasi-experimental design and with the limitations already acknowledged in the manuscript.

**Comment 7. The very large effect sizes deserve slightly deeper interpretation. The authors may briefly consider whether the magnitude of the effects could partly reflect the close alignment between EMKONTAN learning activities and the competencies being assessed, especially for creative thinking and collaboration.**

**Response:** Thank you. We have expanded the Discussion of the very large effect sizes. The revised manuscript now acknowledges that the EMKONTAN activities were closely aligned with several indicators assessed in environmental literacy, creative thinking, and collaboration. Students in the EMKONTAN condition repeatedly practiced environmental investigation, solution generation and refinement, collaborative implementation, and reflection. We therefore clarify that the very large effects may represent both competency development and strong instructional alignment between the learning activities and the assessed competencies. We also note that the magnitude of the effects should not automatically be generalized to other assessment formats, courses, populations, or implementation contexts.

**Comment 8. The interpretation of the collaboration findings could be refined. Since the EMKONTAN group had substantially more opportunities to demonstrate collaborative behavior, the authors may acknowledge that the higher scores could reflect both competency development and greater opportunities to display the assessed behaviors.**

**Response:** We fully agree. The collaboration section has been revised to acknowledge explicitly that the EMKONTAN condition provided substantially more opportunities to practice and demonstrate collaborative behavior than the comparison conditions. We now state that the higher collaboration scores may therefore reflect both development of collaborative competence and greater opportunities to display productive work, compromise, respect, and shared responsibility. We also identify this task–assessment alignment as a possible contributor to the large collaboration effect size.

**Comment 9. The practical implications for pre-service biology teacher education could be made more concrete. The authors may briefly explain how EMKONTAN could be implemented in environmental science, ecology, conservation biology, or teaching-method courses.**

**Response:** Thank you. We have expanded the practical implications to provide concrete examples of implementation. The revised Discussion explains how EMKONTAN could be used in environmental science courses for campus-based mapping of waste, water, vegetation, or pollution; in ecology and conservation biology courses for biodiversity mapping, habitat observation, restoration planning, or conservation campaigns; and in biology teaching-method courses for developing lesson plans, field-learning scenarios, student projects, and assessment tasks. We also emphasize the value of the follow-up phase for helping pre-service teachers transform one-time environmental activities into more sustainable educational programs.

**Comment 10. A short synthesis paragraph at the end of the Discussion would improve the manuscript. Rather than repeating the statistical results, the authors could summarize the main pedagogical contribution of the study, particularly how the integration of environmental inquiry, authentic action, collaboration, monitoring, and follow-up may support sustainability-oriented teacher education.**

**Response:** We agree and have added a synthesis paragraph at the end of the Discussion. Rather than repeating the statistical results, the new paragraph emphasizes that the main pedagogical contribution of EMKONTAN lies in integrating environmental inquiry, authentic action, collaborative participation, monitoring, evaluation, and follow-up within one continuous learning cycle. It also clarifies how this integrated pathway may connect sustainability competencies with the pedagogical preparation needed by future biology teachers.

 We appreciate the reviewer’s comments, which have helped us substantially strengthen the theoretical rationale, positioning of the EMKONTAN model, interpretation of the findings, and practical contribution of the manuscript.

# Reviewer report: Reviewer response for version 1

**DOI:** 10.5256/f1000research.200807.r488172

**Published:** 2026-06-03

**Authors:** Nasrul Hakim

**Affiliations:** 1 State Islamic Institute Metro, Metro, Indonesia

- The PBL and regular instruction conditions should be described in enough detail to allow meaningful comparison.
- Tables and figures are useful, but the text sometimes repeats the same numerical results. The authors could streamline the Results section.
- The Discussion should include a more balanced consideration of alternative explanations for the large observed effects.
- The limitations section is helpful but should be strengthened and linked more directly to the interpretation of the results.

Recommendation

 I recommend that the article undergo minor revision before approval. The manuscript addresses an important and timely topic and has potential value for sustainability education and biology teacher preparation. However, several presentation and reporting issues should be addressed to improve clarity, consistency, and readability. In particular, the authors should describe the PBL and regular instruction conditions in greater detail, streamline repeated numerical reporting in the Results section, provide a more balanced discussion of possible alternative explanations for the large observed effects, and strengthen the limitations section by linking it more explicitly to the interpretation and generalizability of the findings.

Is the work clearly and accurately presented and does it cite the current literature?

Yes

If applicable, is the statistical analysis and its interpretation appropriate?

Yes

Are all the source data underlying the results available to ensure full reproducibility?

Partly

Is the study design appropriate and is the work technically sound?

Yes

Are the conclusions drawn adequately supported by the results?

Yes

Are sufficient details of methods and analysis provided to allow replication by others?

Partly

Reviewer Expertise:

biology, sustainability

I confirm that I have read this submission and believe that I have an appropriate level of expertise to confirm that it is of an acceptable scientific standard.

# Response

**Published:** 2026-06-05

**Authors:** Nurwidodo Nurwidodo

**Affiliations:** Biology Education, Universitas Muhammadiyah Malang, Malang, East Java, Indonesia

Dear Reviewer 2,

 We sincerely thank the reviewer for the thoughtful and constructive comments on our manuscript. We appreciate the reviewer’s recognition that the study addresses an important and timely topic in sustainability education and biology teacher preparation. We have carefully revised the manuscript to improve clarity, consistency, and readability, particularly by expanding the description of the PBL and regular instruction conditions, streamlining repeated numerical reporting in the Results section, adding alternative explanations for the large observed effects, and strengthening the limitations section.

 Comment 1

 The PBL and regular instruction conditions should be described in enough detail to allow meaningful comparison.

**Response:**

 Thank you for this important suggestion. We revised the Learning treatments section to provide a more detailed description of the PBL and regular instruction conditions. The PBL condition is now described as involving problem scenarios, group problem analysis, formulation of learning issues, independent information search, group discussion, solution development, presentation, and reflection. The regular instruction condition is now described as involving lecturer explanation, textbook-based learning, question-and-answer sessions, individual assignments, and conventional assessment. We also clarified that, unlike EMKONTAN, the PBL condition did not require structured environmental mapping, direct environmental action implementation, monitoring, or follow-up program design.

**Revision made in the manuscript:**

 We expanded the description of the comparison groups to make the contrast among EMKONTAN, PBL, and regular instruction more transparent and replicable.

 Comment 2

 Tables and figures are useful, but the text sometimes repeats the same numerical results. The authors could streamline the Results section.

**Response:**

 Thank you for this helpful comment. We revised the Results section to reduce unnecessary repetition of numerical information already presented in the tables and figures. The revised text now focuses more on the main patterns, comparative interpretation, and practical meaning of the results, while leaving detailed values in the tables and figures. We retained key statistical results necessary for interpretation but avoided repeating the same adjusted means, F values, and effect sizes multiple times.

**Revision made in the manuscript:**

 We streamlined the Results section by shortening repetitive numerical descriptions and emphasizing the overall pattern that EMKONTAN consistently outperformed PBL and regular instruction across the three measured outcomes.

 Comment 3

 The Discussion should include a more balanced consideration of alternative explanations for the large observed effects.

**Response:**

 Thank you for this important recommendation. We revised the Discussion to include a more balanced interpretation of the large observed effects. In addition to explaining the pedagogical strengths of EMKONTAN, we added possible alternative explanations, including differences in task structure, student motivation, novelty effects, intensity of group engagement, and the possibility that action-based activities created more opportunities for students to demonstrate the assessed competencies. We also acknowledged that the quasi-experimental design using intact classes prevents full exclusion of unmeasured class-level differences.

**Revision made in the manuscript:**

 We added a paragraph in the Discussion stating that the large effects should not be attributed solely to the intrinsic superiority of EMKONTAN. They may also reflect the alignment between the learning activities and the assessment indicators, increased student engagement due to novelty and authentic tasks, and class-level differences that could not be fully controlled.

 Comment 4

 The limitations section is helpful but should be strengthened and linked more directly to the interpretation of the results.

**Response:**

 Thank you. We strengthened the limitations section by linking each limitation more explicitly to how the findings should be interpreted. We clarified that the use of intact classes limits causal inference, the exclusion of incomplete cases may introduce attrition bias, assumption violations require cautious interpretation of ANCOVA results, and the immediate posttest design limits conclusions about long-term retention and transfer. We also added that the findings are promising but should be generalized carefully to other institutions, student populations, and course contexts.

**Revision made in the manuscript:**

 We revised the limitations section to directly connect methodological limitations with interpretation, generalizability, and future research directions.

 Comment 5

 Are sufficient details of methods and analysis provided to allow replication by others? Partly.

**Response:**

 Thank you for identifying this issue. To improve replicability, we expanded the Learning treatments section, especially the description of the PBL and regular instruction conditions. We also clarified that the research instruments, scoring rubrics, learning treatment descriptions, and supporting materials are available in the extended data. These revisions are intended to provide readers with sufficient methodological detail to understand and replicate the study procedures.

 Comment 6

 Are all the source data underlying the results available to ensure full reproducibility? Partly.

**Response:**

 Thank you for this comment. We revised the Data availability section to make the availability of the underlying and extended data clearer. The dataset includes anonymized pretest and posttest scores, group allocation data, values used to calculate descriptive statistics, adjusted means, gain scores, ANCOVA results, post hoc comparisons, and figure/table data. The extended data include instruments, scoring rubrics, learning treatment descriptions, and supporting materials. This clarification was added to improve transparency and reproducibility.

 Overall response to Reviewer 2

 We are grateful for Reviewer 2’s constructive suggestions. In response, we expanded the description of the comparison conditions, streamlined the Results section, added a more balanced discussion of alternative explanations, strengthened the limitations section, and clarified the availability of underlying and extended data. These revisions have improved the clarity, replicability, and interpretive balance of the manuscript.

# Reviewer report: Reviewer response for version 1

**DOI:** 10.5256/f1000research.200807.r488173

**Published:** 2026-06-02

**Authors:** Ahmad Adnan Mohd Shukri

**Affiliations:** 1 Universiti Sains Malaysia, Penang, Malaysia

1. The study directly addresses a pedagogical gap in sustainability education by introducing the EMKONTAN model, which is well-defined and systematically explained.

 2. The quasi-experimental design with pretest–posttest control groups provides credible comparative evidence. The inclusion of three groups (EMKONTAN, PBL, and regular instruction) strengthens validity.

 3. Environmental literacy, creative thinking, and collaboration were measured using adapted, rubric-based instruments with clear indicators.

 4. Results show large effect sizes (partial eta squared values above 0.70) indicating strong practical significance.

 5. The study offers a contextual instructional model that can be adapted in teacher education programs, especially in developing countries.

 6. **TAKE NOTE:** Only 108 of 120 students were analyzed. The reasons for missing data are not fully explained, which may raise questions about bias.

 7. **TAKE NOTE: **Normality was not met, and collaboration showed heterogeneity of variance. While ANCOVA was applied, results should be interpreted cautiously. **Suggestions:** Justify why ANCOVA was still used (robustness in large samples, exploratory purpose). Highlight that despite assumption violations, the effect sizes were very large, which supports practical significance OR present sensitivity analyses (for example, compare ANCOVA with non-parametric results) to show consistency.

Is the work clearly and accurately presented and does it cite the current literature?

Yes

If applicable, is the statistical analysis and its interpretation appropriate?

Yes

Are all the source data underlying the results available to ensure full reproducibility?

Yes

Is the study design appropriate and is the work technically sound?

Yes

Are the conclusions drawn adequately supported by the results?

Yes

Are sufficient details of methods and analysis provided to allow replication by others?

Yes

Reviewer Expertise:

STEM education; biology education; design and development research; challenge-based learning; computational thinking skills; creative thinking skills and styles; critical thinking skills and dispositions

I confirm that I have read this submission and believe that I have an appropriate level of expertise to confirm that it is of an acceptable scientific standard.

# Response

**Published:** 2026-06-05

**Authors:** Nurwidodo Nurwidodo

**Affiliations:** Biology Education, Universitas Muhammadiyah Malang, Malang, East Java, Indonesia

Dear Reviewer 1,

 We sincerely thank the reviewer for the careful and constructive evaluation of our manuscript. We are grateful for the positive comments regarding the relevance of the EMKONTAN model, the quasi-experimental design, the use of three comparison groups, the rubric-based instruments, the large practical effects, and the contextual contribution of the study to sustainability-oriented teacher education. We have carefully revised the manuscript in response to the reviewer’s notes, especially regarding the explanation of missing data and the interpretation of ANCOVA under assumption violations.

 Comment 1

 The study directly addresses a pedagogical gap in sustainability education by introducing the EMKONTAN model, which is well-defined and systematically explained.

**Response:**

 Thank you for this positive assessment. We are pleased that the reviewer recognized the pedagogical contribution of the EMKONTAN model. In the revised manuscript, we retained and strengthened the explanation of EMKONTAN as an environmental action-based learning cycle consisting of problem identification, environmental observation, action planning, implementation, monitoring and evaluation, and follow-up program design. This explanation is intended to clarify how the model addresses the gap between problem-oriented learning and concrete sustainability action.

 Comment 2

 The quasi-experimental design with pretest–posttest control groups provides credible comparative evidence. The inclusion of three groups (EMKONTAN, PBL, and regular instruction) strengthens validity.

**Response:**

 Thank you for this supportive comment. We agree that the inclusion of EMKONTAN, PBL, and regular instruction provides a meaningful comparative structure. In the revised manuscript, we clarified the role of each group: EMKONTAN as the environmental action-based intervention, PBL as the positive control representing a problem-oriented approach, and regular instruction as the negative control representing conventional teaching. This revision was made to improve transparency and comparability across the three learning conditions.

 Comment 3

 Environmental literacy, creative thinking, and collaboration were measured using adapted, rubric-based instruments with clear indicators.

**Response:**

 Thank you. We retained the description of the instruments and indicators in the revised manuscript and ensured that the measurement of environmental literacy, creative thinking, and collaboration remained clearly aligned with the intended sustainability competencies. We also clarified that the instruments and rubrics are available as extended data to support transparency and replication.

 Comment 4

 Results show large effect sizes, indicating strong practical significance.

**Response:**

 Thank you for highlighting this point. In the revised manuscript, we retained the reporting of partial eta squared values and emphasized that the large effect sizes indicate practical significance. However, in response to the reviewer’s later statistical note, we also revised the interpretation to be more cautious by acknowledging that the large effect sizes should be interpreted alongside the quasi-experimental design and the assumption-test results.

 Comment 5

 The study offers a contextual instructional model that can be adapted in teacher education programs, especially in developing countries.

**Response:**

 We appreciate this comment. In the revised Discussion and Conclusion, we strengthened the explanation of the contextual contribution of EMKONTAN for biology teacher education, particularly in developing-country higher education settings. We emphasized that the model provides pre-service teachers with an instructional repertoire for designing participatory, action-oriented environmental learning.

 Comment 6

 Only 108 of 120 students were analyzed. The reasons for missing data are not fully explained, which may raise questions about bias.

**Response:**

 Thank you for this important observation. We revised the Participants and setting section to explain more clearly why only 108 of the 120 eligible students were included in the final analysis. We clarified that complete-case analysis was applied because 12 students had incomplete pretest or posttest records due to absence from one of the testing sessions or incomplete submission of one or more assessment components. We also added a statement acknowledging that excluding incomplete cases may introduce attrition-related bias and that this issue should be considered when interpreting the findings.

**Revision made in the manuscript:**

 We added a clearer explanation that the final sample consisted of students with complete pretest and posttest data across all measured variables, while 12 students were excluded due to incomplete assessment records. We also acknowledged this as a limitation.

 Comment 7

 Normality was not met, and collaboration showed heterogeneity of variance. While ANCOVA was applied, results should be interpreted cautiously. Suggestions: Justify why ANCOVA was still used or present sensitivity analyses.

**Response:**

 Thank you for this valuable statistical comment. We revised the Data analysis, Results, Discussion, Limitations, and Conclusion sections to provide a more cautious interpretation of the ANCOVA findings. We clarified that ANCOVA was retained because the study involved a balanced educational comparison with a complete-case sample of 108 students, ANCOVA is commonly used to control baseline differences in quasi-experimental pretest–posttest designs, and the analysis was intended to compare adjusted posttest means while controlling for pretest scores. At the same time, we explicitly acknowledged that the violation of normality and the heterogeneity of variance for collaboration require cautious interpretation. We also added that future studies should complement ANCOVA with robust or rank-based covariance procedures and sensitivity analyses.

**Revision made in the manuscript:**

 We added a cautionary statement explaining that the results should be interpreted as statistically informative but not definitive, particularly for collaboration. We also emphasized that the very large effect sizes and consistent descriptive patterns support the practical significance of the findings, while future studies should test the robustness of the results using complementary non-parametric or robust methods.

 Overall response to Reviewer 1

 We thank Reviewer 1 for the encouraging evaluation and constructive suggestions. The manuscript has been revised to improve transparency regarding missing data, strengthen the justification and cautious interpretation of ANCOVA, and clarify the methodological limitations related to assumption violations and complete-case analysis.
